# Intestinal *Fut2* deficiency exacerbated alcohol‐related liver disease by disrupting nicotinamide metabolism

**DOI:** 10.1002/ctm2.70447

**Published:** 2025-08-15

**Authors:** Liuying Chen, Zhongwei Yin, Luorui Shang, Hang Yuan, Wenkang Gao, Shuhan Wang, Shuyan Wang, Xiaohua Hou, Shenglan Yang, Huikuan Chu

**Affiliations:** ^1^ Department of Clinical Nutrition Union Hospital Tongji Medical College Huazhong University of Science and Technology Wuhan China; ^2^ Division of Gastroenterology Union Hospital Tongji Medical College Huazhong University of Science and Technology Wuhan China; ^3^ Division of Cardiology and Hubei Key Laboratory of Genetics and Molecular Mechanisms of Cardiological Disorders Tongji Hospital Tongji Medical College Huazhong University of Science and Technology Wuhan China

**Keywords:** alcohol‐related liver disease, fucosyltransferase 2, NAD, nicotinamidase

## Abstract

**Background:**

Fucosyltransferase 2 (FUT2)‐dependent fucosylation of intestinal epithelial cells is vital for preserving gut barrier integrity and microbial balance. Nevertheless, its precise involvement in alcohol‐associated liver disease has yet to be fully elucidated.

**Methods:**

We generated mice with intestinal epithelial cell‐specific *Fut2* knockout (*Fut2* ^△IEC^) and established a chronic‐binge alcohol model. 16S rRNA sequencing and metabolomics analysis were used to reveal differences in the composition and function of faecal bacteria.

**Results:**

The loss of intestinal epithelial *Fut2* exacerbates alcohol‐related hepatic oxidative stress damage, and this effect is dependent on gut bacteria. A marked decrease in the abundance of bacteria carrying nicotinamidase (PncA) in the intestines of *Fut2* ^△IEC^ mice was observed, leading to disrupted nicotinamide metabolism and decreased nicotinic acid production. This reduction in nicotinic acid synthesis results in decreased NAD^+^ production in the liver via the Preiss–Handler pathway. Administering *pncA*‐overexpressing *Escherichia coli* promotes hepatic NAD^+^ synthesis and alleviates alcohol‐related oxidative stress damage in *Fut2* ^△IEC^ mice.

**Conclusion:**

These findings reveal a gut microbiota–*Fut2*–*pncA* axis that modulates alcoholic liver injury in mice, which may offer insights into microbial contributions to alcoholic liver disease in people.

**Key points:**

- Loss of intestinal epithelial fucosylation exacerbates alcohol‐related liver injuries.
- Intestinal epithelial deletion of *Fut2* disrupts nicotinamide (NAM) metabolism due to the decline in bacterial nicotinamidase (PncA).
- Supplemented with *pncA* overexpressed *E.coli* to restore gut PncA levels can improve liver damage in alcohol‐induced intestinal epithelial *Fut2* deletion mice.

## Graphical abstract

1. Loss of intestinal epithelial fucosylation exacerbates alcohol‐related liver injuries.
2. Intestinal epithelial deletion of *Fut2* disrupts nicotinamide (NAM) metabolism due to the decline in bacterial nicotinamidase (PncA).
3. Supplemented with *pncA* overexpressed *E.coli* to restore gut PncA levels can improve liver damage in alcohol‐induced intestinal epithelial *Fut2* deletion mice.

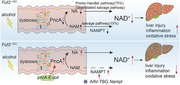

## 1 INTRODUCTION

Alcohol consumption is prevalent worldwide, with 43% of the population currently consuming alcohol.^1^ Alcohol intake disrupts the intestinal epithelial barrier, leading to increased gut permeability, which is now recognised as a significant factor in alcohol‐related liver disease (ALD) development.^2^ In alcohol‐fed mice, sensitivity to ALD appears to be influenced by the composition of intestinal microbiota.^3^, ^4^ Fucosyltransferases (FUTs) play distinct roles in intestinal fucosylation by catalysing the addition of fucose residues through specific glycosidic linkages. FUT1 and FUT2 are α1,2‐fucosyltransferases responsible for synthesising H antigen and Lewis b structures; among them, FUT2 is predominantly expressed in intestinal epithelial cells and is vital for modulating the gut microbiota and maintaining mucosal barrier integrity.^5^ FUT3–FUT7 mediates α1,3‐ or α1,4‐fucosylation, leading to the formation of Lewis a, Lewis x and sialyl‐Lewis x. These fucosylated glycans act as metabolic substrates, energy sources and adhesion sites for various beneficial symbiotic bacteria.^6^, ^7^ Previous studies have indicated that the fucosyltransferase 2 gene (*Fut2*) knockout increases susceptibility to alcoholic liver injury in mice by altering intestinal microbiota.^8^

Individuals with variants in the *FUT2* gene, known as non‐secretors, were found to be resistant to *Rotaviruses*, *Norovirus*, *Sapovirus* and *Campylobacter jejuni*, but susceptible to *Escherichia coli*.^9^ Genome‐wide association studies (GWAS) have revealed associations between *FUT2* secretor status and *Bacteroides* and *Faecalibacterium* species.^10^ Furthermore, *FUT2* loss‐of‐function variants contribute to a greater risk of primary sclerosing cholangitis (PSC) and liver diseases.^11^, ^12^ How intestinal α1‐2‐fucosylation regulates gut bacteria to aggravate alcohol‐related liver disease is unclear.

Nicotinamide adenine dinucleotide (NAD^+^) is a co‐substrate of several enzymes and plays an important role in mitochondrial homeostasis, metabolism and lifespan of organisms.^13^ A decrease in the cellular NAD^+^ pool triggered alcohol‐related hepatic inflammation and steatosis.^14^ Supplementation of the NAD^+^ precursor,^15^, ^16^ nicotinamide riboside, or restoring the NAD^+^/NADH ratio^17^ could attenuate liver inflammation and oxidative stress in experimental alcoholic liver injuries in mice. NAD⁺ synthesis occurs through three main pathways: the de novo pathway starting from tryptophan, the salvage pathway utilising nicotinamide (NAM), and the Preiss–Handler pathway involving nicotinic acid (NA). In the salvage pathway, NAM is converted into nicotinamide mononucleotide (NMN) by nicotinamide phosphoribosyltransferase (NAMPT), which is then converted to NAD⁺ by NMN adenylyltransferase (NMNAT). The Preiss–Handler pathway begins with the conversion of NA to nicotinic acid mononucleotide (NaMN) by nicotinic acid phosphoribosyltransferase (NAPRT), followed by transformation into nicotinic acid adenine dinucleotide (NaAD) via NMNAT, and finally amidation to NAD⁺ catalysed by NAD⁺ synthetase (NADSYN).^18^ The *pncA* encodes nicotinamidase, which enables gut bacteria to convert nicotinamide (NAM) into NA, thereby fueling host NAD^+^ production via the Preiss–Handler route.^19^ In this study, we found that the absence of intestinal α1,2‐fucosylation impaired hepatic NAD⁺ synthesis through the Preiss–Handler pathway. Consequently, alcohol‐related liver injury in *Fut2* ^△IEC^ mice was mitigated by administration of *pncA*‐overexpressing *E. coli*.

## 2 MATERIALS AND METHODS

### 2.1 Clinical samples

Colonic biopsy and plasma samples were obtained from individuals with alcohol misuse, alcoholic hepatitis, and from healthy controls. This study enrolled individuals aged over 18 years, categorised into three groups: ALD patients diagnosed according to the 2018 Chinese guideline with a history of heavy alcohol use; individuals with chronic alcohol consumption but no liver injury (pure drinking group); and healthy controls without alcohol use or liver disease. Exclusion criteria included liver malignancies, infectious or non‐infectious liver diseases, severe comorbidities in other organs, and pregnancy or lactation.

### 2.2 Animal experiments

Villin‐Cre transgenic C57BL/6 mice were crossed with *Fut2* floxed mice (purchased from GemPharmatech Co. Ltd.) to generate intestinal epithelial cell‐specific *Fut2* knockout mice (Villin‐Cre *Fut2* ^flox/flox^, hereafter referred to as *Fut2* ^△IEC^ mice). Transgenic mice were validated through qPCR‐based genotyping and Ulex europaeus agglutinin‐I (UEA‐1) immunofluorescence staining of the colon (Figure S1). *Fut2* ^flox/flox^ mice without the Pvillin‐Cre recombinase were used as controls (wild‐type [WT]). Male mice aged 8–10 weeks were used to establish chronic‐binge alcoholic liver disease models.^20^ After a 5‐day adaptation to the liquid diet, the mice were fed a Lieber–De Carli diet containing 5% (v/v) ethanol for 10 days. Finally, they received a binge dose of 5 g of ethanol per kg bodyweight.^21^ The control group was given an isocaloric maltodextrin‐based diet. Samples were harvested 9 h after binging.

For the *pncA* treatment experiment, *Fut2* ^△IEC^ mice were gavaged with 2 × 10^8^ WT or *pncA*‐overexpressing *E. coli* (*pncA E. coli*) daily while fed an alcoholic liquid diet.

### 2.3 Liver histopathology and biochemical assays

Paraffin‐embedded liver tissues were subjected to haematoxylin and eosin (H&E) staining according to standard protocols. Liver samples embedded in OCT were sliced into 7 µm sections and stained with Oil Red O (Sigma‐Aldrich). Serum alanine aminotransferase (ALT), aspartate aminotransferase (AST) and ethanol levels were analysed by commercial kits (Nanjing Jiancheng Institute of Bioengineering).

### 2.4 Immunohistochemistry (IHC) and immunofluorescence (IF)

Liver sections were stained using a goat anti‐rabbit IgG (H+L) IHC kit (Servicebio). Primary antibodies against FUT2 (1:200; Immumoway) and 4‐hydroxynonenal (4‐HNE) (1:200; GeneTex) were used. For IF staining, colonic tissues were incubated with UEA‐I conjugated to DyLight 649 (1:200; Vector Laboratories).

### 2.5 Reverse transcription‑polymerase chain reaction (RT‑PCR)

Total RNA was extracted from liver tissues of *Fut2* ^△IEC^ and WT mice with control or alcohol diets, and quantified following standard protocols. Gene‐specific primers targeting *Il‐1β*, *Il‐6*, *Mcp‐1*, *Tnf‐α*, *Tgf‐β*, *Nrf2*, *Gpx1*, *Gpx4*, *Sod2*, *Naprt*, *Nmnat1*, *Nmnat3*, *Nadsyn* and *Nampt* were used. Relative mRNA levels were determined by the 2^–ΔΔCt^ method. Primer sequences are provided in Table S1.

### 2.6 Western blotting

Mouse liver tissue samples were collected, homogenised and centrifuged to extract the cellular supernatant. Proteins were separated and transferred according to standard protocols, and detected using an anti‐NAMPT antibody (1:500; Proteintech).

### 2.7 16S rRNA sequencing

Faecal DNA was extracted from *Fut2* ^△IEC^ and WT mice. The V3–V4 hypervariable regions of the 16S rRNA gene were amplified and sequenced using the Illumina NovaSeq platform (Wekemo Tech Co., Ltd.). QIIME software was employed to generate operational taxonomic unit (OTU) abundance tables for each sample. Alpha diversity of the gut microbiota was assessed by Chao1, Shannon and observed indices, while beta diversity was evaluated through principal coordinate analysis (PCoA). Differential bacterial taxa from phylum to genus levels were identified using linear discriminant analysis effect size (LEfSe).

### 2.8 Untargeted metabolome analysis

Stool samples from *Fut2* ^△IEC^ and WT mice, along with plasma samples from healthy controls (HC), individuals with alcohol misuse (AM), and patients with alcoholic hepatitis (AH), were evaluated by high‐performance liquid chromatography‐mass spectrometry (LC‐MS). Original datasets underwent quality control and normalisation before analysis. Principal component analysis (PCA) was conducted to identify notable variations among groups. Differential metabolites between *Fut2* ^△IEC^ and WT mice were identified. KEGG pathway enrichment of these metabolites was assessed through over‐representation analysis.

### 2.9 Construction of *E. coli* overexpressing *pncA* and mice treatment

The *pncA* gene was cloned into a modified pET‐15b plasmid with ampicillin resistance using the XhoI and BamHI sites. The cloned *pncA* sequence was confirmed by Sanger sequencing, subsequently inserted into competent *E. coli* BL21 (DE3) cells to generate *pncA*‐overexpressing strains (referred to as *pncA E. coli*). As a control, an empty plasmid was inserted into *E. coli* BL21 (DE3). Gene expression was induced by 1 mM isopropyl‐β‐D‐thiogalactopyranoside (IPTG). For the *pncA* treatment experiment, *Fut2* ^△IEC^ mice were gavaged with 2 × 10^8^ WT or *pncA E. coli* (resuspended in fresh culture LB medium with IPTG) daily for 10 days while fed an alcoholic liquid diet.

### 2.10 Faecal DNA extraction and amplification

Faecal DNA was extracted using a commercial stool DNA extraction kit (TIANGEN) from *Fut2* ^△IEC^ mice, WT mice and *Fut2* ^△IEC^ mice exposed to ethanol and treated with either WT *E. coli* or *pncA E. coli*. Primer sequences are listed in Table S2.

### 2.11 NAD^+^/NADH measurement in liver

Quantification of hepatic NAD⁺ and NADH was performed following the protocol provided with the commercial assay kit (Beyotime Biotechnology).

### 2.12 Adeno‐associated virus (AAV) and infection

The full‐length cDNA of the mouse *Nampt* gene was cloned into an adeno‐associated virus serotype 8 (AAV8) vector under the control of the hepatocyte‐specific thyroglobulin (TGB) promoter (Genechem). The AAV8‐*Nampt* virus was produced by transfection of HEK293T cells using the AAV helper plasmids and purified by iodixanol gradient ultracentrifugation. AAV‐TBG *Nampt* and AAV‐TBG empty vectors (2 × 10^11^ vg/mouse) were delivered to *Fut2* ^△IEC^ mice via a single tail vein injection. Two weeks later, the mice were fed the Lieber–De Carli diet.

### 2.13 Statistical analysis

Results are presented as mean ± standard error of the mean (SEM). Statistical differences between two groups were assessed using a two‐sided unpaired Student's *t*‐test or the Mann–Whitney *U* test, as appropriate. For comparisons among multiple groups, two‐way ANOVA followed by Tukey's post hoc test was performed. Statistical significance was defined as *p* < .05.

## 3 RESULTS

### 3.1 Lack of epithelial α1,2‐fucosylation exacerbated alcohol‐related liver disease

**FIGURE 1:**
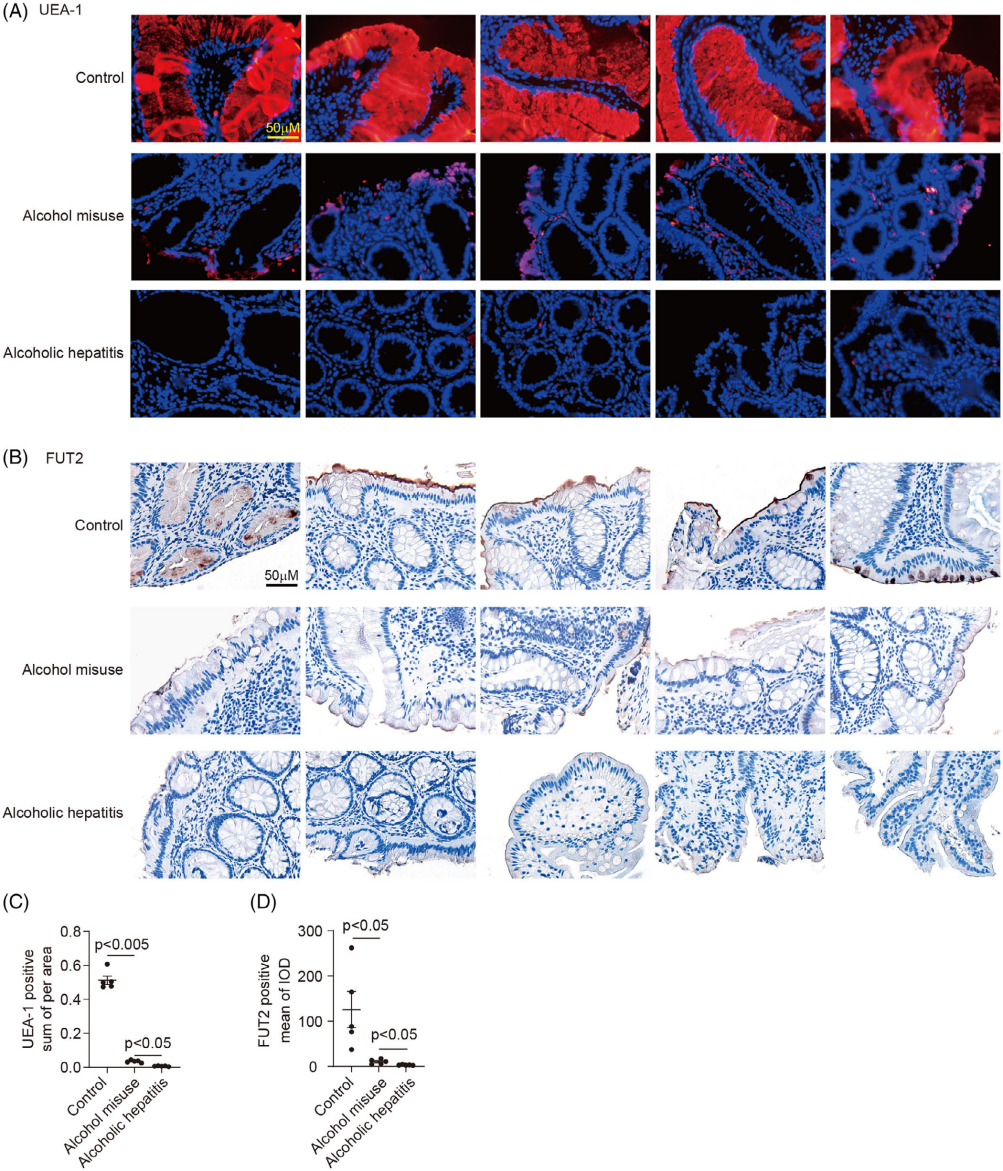
Decreased intestinal α1‐2‐fucosylation in patients with alcoholic liver disease. (A and C) The expression (A) and quantification (C) of intestinal α1‐2 fucosylation of colonic biopsies measured by Ulex europaeus agglutinin I (UEA‐I) staining in control subjects (*n* = 5), patients with alcohol misuse (*n* = 5), and patients with alcoholic hepatitis (*n* = 5). (B and D) The expression (B) and quantification (D) of Fut2 expressions of the above groups.

FUT2‐mediated α1,2‐fucosylation is pivotal to maintain intestinal homeostasis. To investigate its alteration in alcoholic liver disease, we assessed intestinal fucosylation levels in patients. Colonic epithelial fucosylation, indicated by UEA‐I staining, was markedly decreased in individuals with alcohol misuse and alcoholic hepatitis (Figure 1A,C). FUT2 protein expression in the colonic epithelial cells of patients with alcohol misuse and alcoholic hepatitis was markedly decreased compared to normal controls (Figure 1B,D).

**FIGURE 2:**
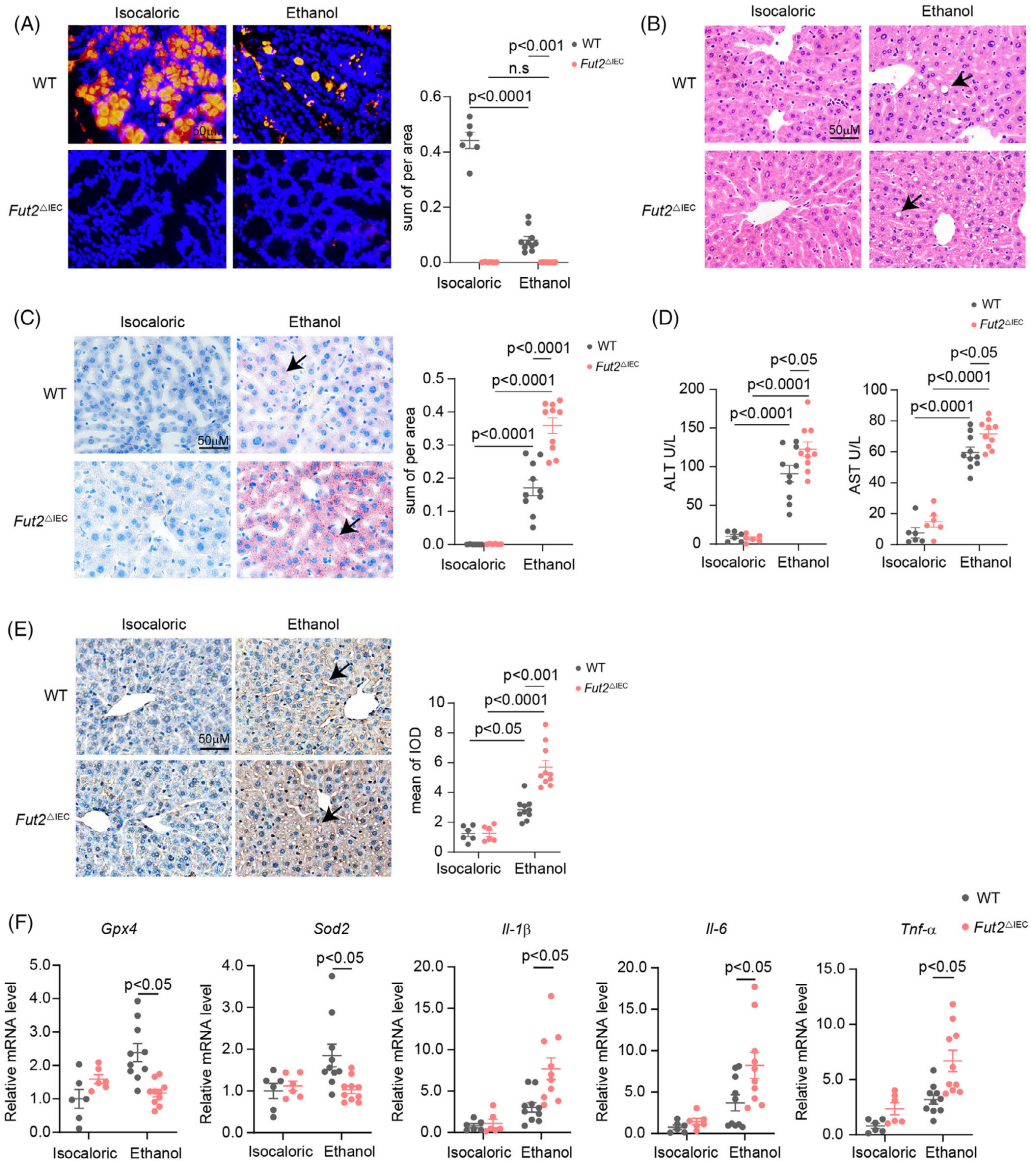
Intestinal epithelial *Fut2* deficiency exacerbated alcohol‐induced liver damage. (A) Colonic UEA‐I staining of control and alcohol‐fed *Fut2^△IEC^* and WT mice. (B) Histological examination of liver tissues from control and alcohol‐fed *Fut2^△IEC^* and WT mice using haematoxylin and eosin (H&E) staining. Arrows indicate lipid droplets. (C) Oil Red O staining of liver tissues of the above groups of mice. Arrows indicate lipid droplets. (D) Levels of ALT and AST in the serum of mice from the above groups. (E) Immunohistochemical staining of 4‐HNE indicates oxidative stress damage in the liver tissues of each group. Arrows indicate positive areas. (F) mRNA levels of oxidative stress‐related enzymes *Gpx4* and *Sod2*, and inflammatory factors *Il‐1β*, *Il‐6* and *Tnf‐α* in the liver of control and alcohol‐fed *Fut2^△IEC^* and WT mice. WT mice and *Fut2^△IEC^* mice exposed to isocaloric food: *n* = 6, WT mice and *Fut2^△IEC^* mice exposed to ethanol food: *n* = 10.

*Fut2* ^△IEC^ mice exhibited a complete loss of colonic epithelial fucosylation, whereas WT mice showed reduced fucosylation following alcohol exposure (Figure 2A). Compared to WT controls, *Fut2* ^△IEC^ mice fed an ethanol‐containing diet developed more severe hepatic steatosis, based on H&E and Oil Red O staining (Figure 2B,C). Serum levels of ALT and AST were significantly elevated in *Fut2* ^△IEC^ mice, indicating greater liver injury (Figure 2D). Although chronic‐binge ethanol feeding increased intestinal permeability in WT as well as *Fut2* ^△IEC^ mice, the two groups showed comparable results (Figure S2A,B). Similarly, serum ethanol concentrations showed comparable results (Figure S2C).

Ethanol exposure induced greater oxidative stress in *Fut2* ^△IEC^ mice, as evidenced by increased hepatic 4‐HNE levels compared to WT mice (Figure 2E). Furthermore, alcohol‐exposed *Fut2* ^△IEC^ mice exhibited a significant reduction in the hepatic mRNA expression of antioxidant enzymes *Gpx4* and *Sod2* (Figure 2F). The inflammatory response was also exacerbated, with higher hepatic expression of *Il‐1β*, *Il‐6* and *Tnf‐α* compared to alcohol‐fed WT controls (Figure 2F).

These findings suggest that *Fut2* deletion in intestinal mucosal cells exacerbates alcohol‐induced hepatic oxidative stress and inflammation.

### 3.2 Mice lacking epithelial α1,2‐fucosylation exhibited intestinal dysbiosis

**FIGURE 3:**
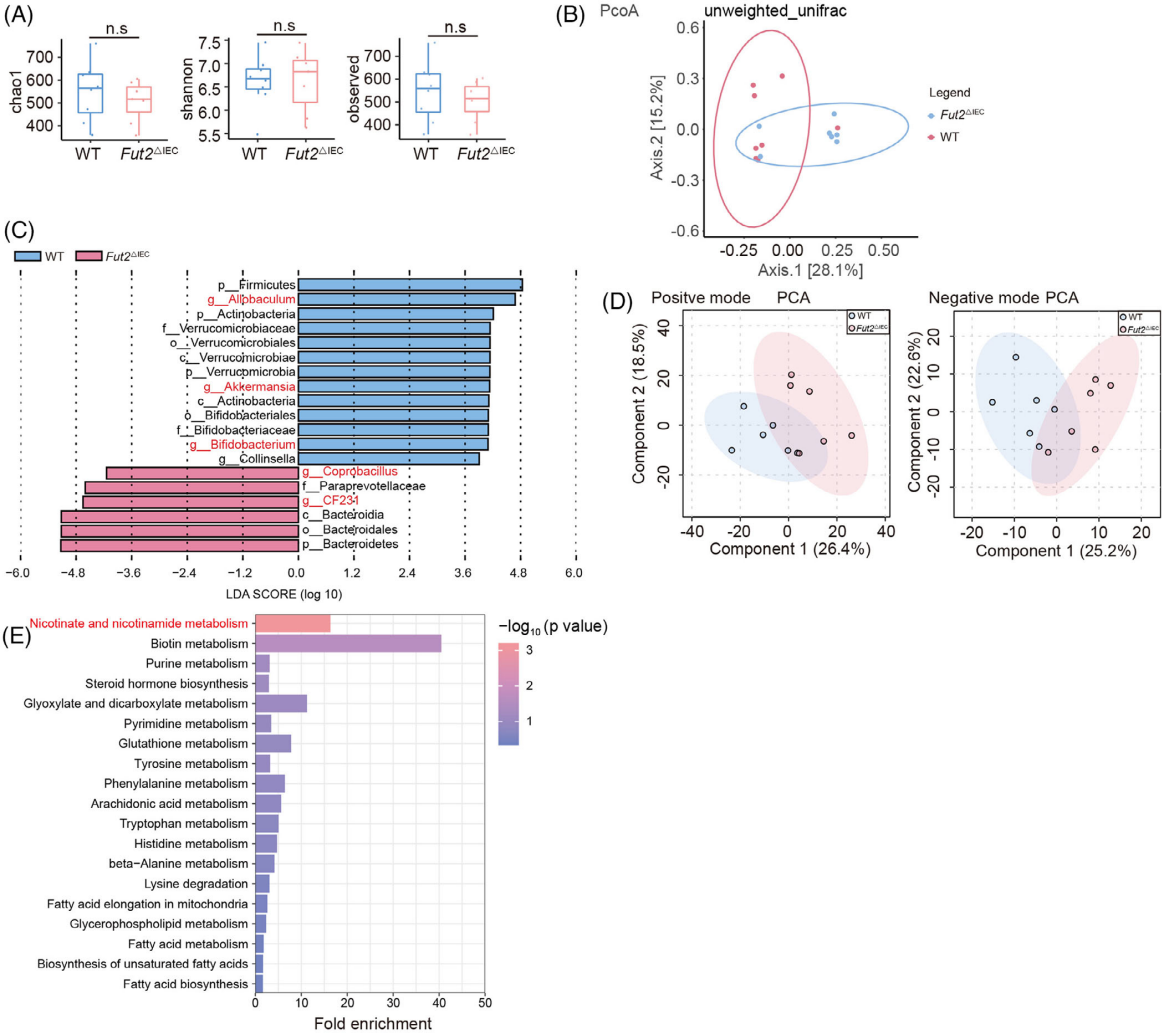
The composition of gut bacteria and metabolites shows significant differences between *Fut2^△IEC^* and WT mice. (A) Differences in bacterial richness and diversity measured by chao1, Shannon, and observed indexes between *Fut2* ^△IEC^ (*n* = 7) and WT (*n* = 8) mice. (B) Differences in bacterial richness and diversity measured by PcoA analysis. (C) List of different bacteria between *Fut2^△IEC^* and WT mice measured by LEfSe. (D) PCA analysis of metabolites between *Fut2^△IEC^* (*n* = 6) and WT (*n* = 6) mice in the positive‐ and negative‐ion modes. (F) KEGG pathways of differential metabolites enrichment. n.s.: no significant difference.

Although the Chao1, Shannon, and observed indices revealed no evident differences in α‐diversity between the two groups (Figure 3A), β‐diversity analysis using principal coordinate analysis (PCoA) revealed a marked separation in microbial community structure (Figure 3B). Notably, *Fut2* ^△IEC^ mice displayed a notable reduction in the relative abundance of *Allobaculum* (*p* = .015), *Akkermansia* (*p* = .025), and *Bifidobacterium* (*p* = .028), with a marked increase in *Coprobacillus* (*p* = .0016) and *CF231* (*p* = .008) compared to WT controls (Figure 3C). The findings demonstrate that epithelial α1,2‐fucosylation is vital to shape the gut microbial composition.

### 3.3 Lack of epithelial α1‐2‐fucosylation regulated gut metabolites

Non‐targeted metabolome sequencing of stool samples from *Fut2^△IEC^* and WT mice PCA (Figure 3D) analyses showed that the metabolomes of *Fut2^△IEC^* mice were clearly distinct from those of the WT mice. In positive ion mode, 28 metabolites were significantly decreased and 59 were elevated in *Fut2^△IEC^* mice versus WT mice (Figure S3). In negative ion mode, a total of seven metabolites showed decreased levels, whereas 34 were elevated in *Fut2^△IEC^* mice. Pathway enrichment analysis identified nicotinate and nicotinamide metabolism as the most significantly altered pathway (Figure 3E). These results indicated that *Fut2* knockout significantly affected bacterial functions, particularly nicotinate and nicotinamide metabolism.

### 3.4 Lack of epithelial α1‐2‐fucosylation decreased nicotinamide deamination by gut bacteria

**FIGURE 4:**
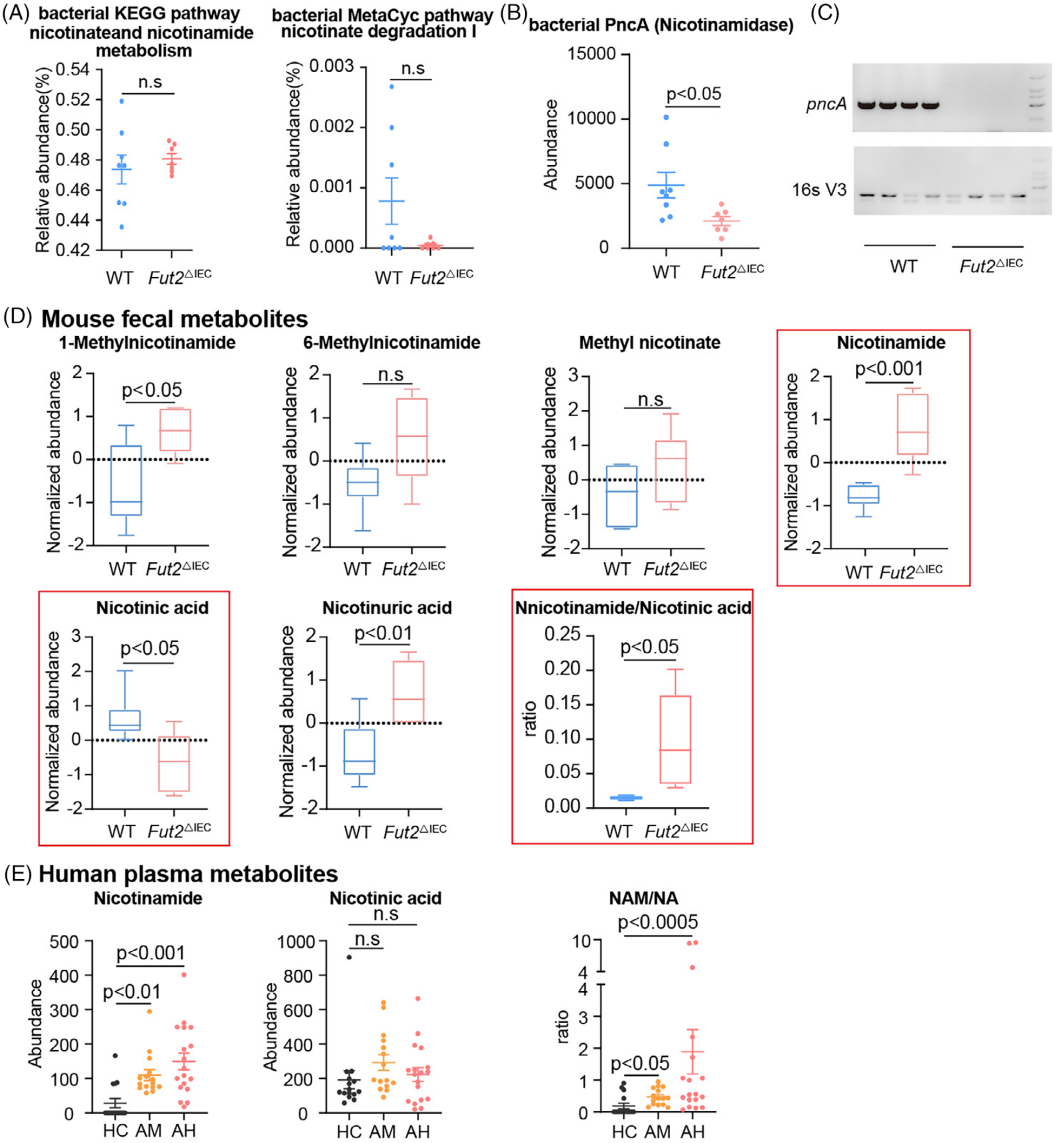
The ability of *Fut2^△IEC^* mice to metabolise nicotinamide into nicotinic acid was diminished. (A) Comparison of nicotinate and nicotinamide metabolism, and nicotinate degradation I signalling pathways in the gut bacteria of *Fut2^△IEC^* mice (*n* = 7) and WT (*n* = 8) mice. (B) Comparison of the abundance of bacterial PncA (nicotinamidase) between *Fut2^△IEC^* (*n* = 7) mice and WT (*n* = 8) mice. (C) Quantitative PCR analysis of *pncA* gene expression in faecal samples from WT and *Fut2^△IEC^* mice (*n* = 4). (D) Comparison of the abundance of nicotinic acid (NA), nicotinamide (NAM), and their metabolites in the gut of *Fut2^△IEC^* mice and WT mice (*n* = 6). (D) Comparison of the abundance of nicotinic acid, nicotinamide, and the ratio of NAM to NA among healthy control (HC) (*n* = 15), alcohol misuse (AM) (*n* = 15) and alcoholic hepatitis (AH) (*n* = 21) patients. n.s.: no significant difference.

Although KEGG pathway analysis showed no differences in bacterial nicotinate and nicotinamide metabolism between *Fut2^△IEC^* mice and WT mice, MetaCyc pathway showed no differences in bacterial nicotinate degradation I between *Fut2^△IEC^* mice and WT mice (Figure 4A), the abundance of nicotinamidase (PncA), but not other related enzymes, was markedly reduced in *Fut2^△IEC^* mice when contrasted with controls (Figure 4B, Figure S4) through 16S rRNA sequencing. This result was further validated by qPCR analysis of faecal samples (Figure 4C). PncA, an enzyme specific to gut microbiota, is responsible for deamidating nicotinamide to produce nicotinic acid; this process ensures that the liver can synthesise NAD^+^ through the Preiss–Handler pathway.^22^

Among metabolites in the nicotinate and nicotinamide metabolism pathways, NAM was significantly elevated, while NA was greatly decreased in *Fut2^△IEC^* mice, in contrast to WT controls (Figure 4D). Additionally, the NAM‐to‐NA ratio was significantly increased in *Fut2^△IEC^* mice, indicating impaired conversion of NAM to NA in these mice.

Similarly, alcohol misuse (AM) and alcoholic hepatitis (AH) patients had a significant increase in the plasma NAM levels and the ratio of NAM to NA, in contrast to healthy controls (HC) (Figure 4E).

### 3.5 Decreased deamination of NAM impaired hepatic NAD^+^ biosynthesis of *Fut2^△IEC^* mice

**FIGURE 5:**
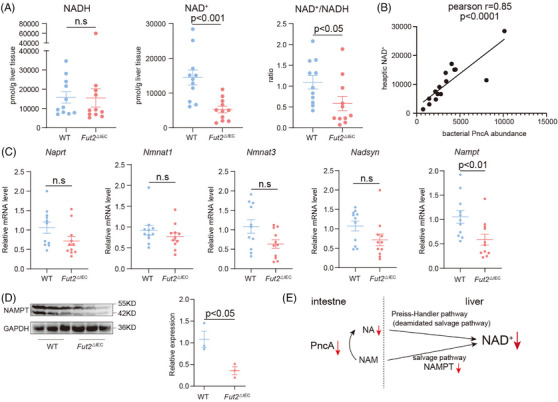
The hepatic NAD^+^ synthesis was impaired in *Fut2^△IEC^* mice. (A) Comparison of hepatic NAD^+^ and NADH levels of *Fut2^△IEC^* mice and WT mice (*n* = 11). (B) Relationship of bacterial PncA abundance and hepatic NAD^+^ measured by Pearson correlation analysis. (C) Expressions of hepatic enzymes involved in the NAD^+^ salvage pathway and Preiss–Handler pathway are compared between *Fut2^△IEC^* mice and WT mice (*n* = 11). (D) NAMPT expression in the liver of *Fut2^△IEC^* mice and WT mice measured by WB (*n* = 3). (E) Schematic diagram of possible pathways affecting NAD^+^ synthesis in the liver of *Fut2^△IEC^* mice. n.s.: no significant difference.

As NAM and NA serve as precursors for hepatic NAD⁺ synthesis, we evaluated liver levels of NAD⁺ and enzymes associated with NAD⁺ biosynthesis in *Fut2^△IEC^* mice. While hepatic NADH levels were comparable between *Fut2^△IEC^* and WT mice (Figure 5A), NAD⁺ levels and the NAD⁺/NADH ratio were significantly reduced in *Fut2^△IEC^* mice. Additionally, faecal PncA abundance positively correlated with hepatic NAD⁺ content (Figure 5B).

Among the hepatic genes involved in salvage pathway NAD^+^ synthesis, *Nampt*, the limiting enzyme, was downregulated in *Fut2^△IEC^* mice (Figure 5C). No meaningful differences were detected in the hepatic expression of Preiss–Handler pathway enzymes, including *Naprt*, *Nmnat1*, *Nmnat3* and *Nadsyn*, between *Fut2^△IEC^* mice and WT mice (Figure 5C). Western blotting provided additional validation of reduced NAMPT expression in *Fut2^△IEC^* mice (Figure 5D).

This indicates that the reduction in hepatic NAD**^+^** synthesis in *Fut2^△IEC^* mice was influenced by two main factors: (i) a decrease in intestinal NA production, and (ii) lowered expression of an important enzyme in the hepatic salvage pathway (Figure 5E).

### 3.6 Restoring NAM salvage pathway synthesis of NAD^+^ in the liver did not alleviate alcoholic liver injury in *Fut2^△IEC^* mice

**FIGURE 6:**
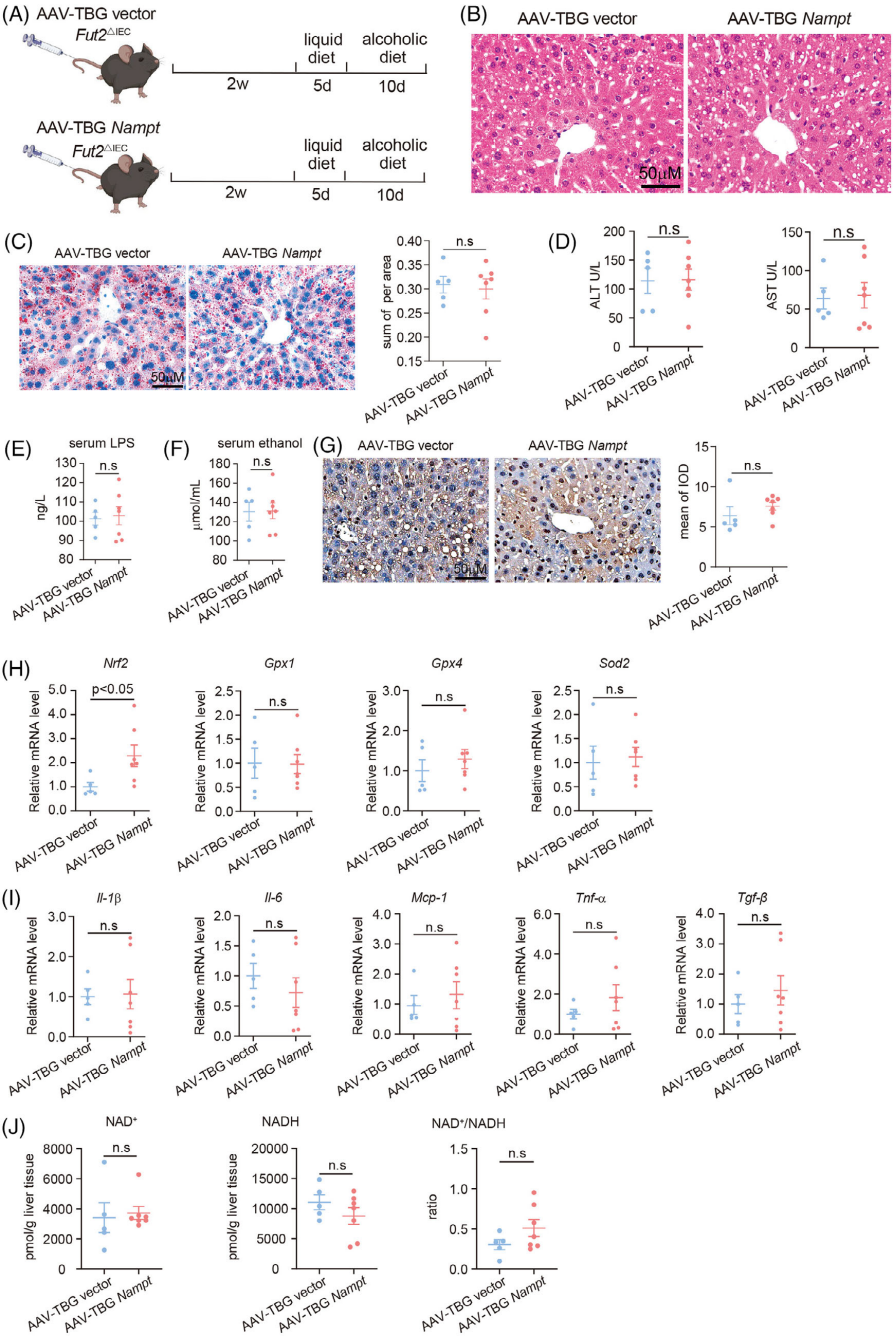
Overexpression of NAMPT in hepatocytes did not ameliorate alcohol‐induced liver damage in *Fut2^△IEC^* mice. (A) The strategy for constructing an alcohol‐induced liver disease model in *Fut2^△IEC^* mice with hepatocyte overexpression of NAMPT. (B and C) Haematoxylin and eosin (H&E) staining (B) and Oil Red O staining (C) of liver tissues from *Fut2^△IEC^* mice with and without hepatocyte overexpression of NAMPT. (D) Comparison of serum ALT and AST levels between the above two groups of mice. (E and F) Comparison of serum LPS (E) and ethanol (F) levels between the above two groups of mice. (G) Immunohistochemical staining of 4‐HNE was conducted to assess oxidative stress damage in the liver tissues of *Fut2^△IEC^* mice with and without hepatocyte overexpression of NAMPT. (H) Levels of oxidative stress‐related enzymes *Nrf2, Gpx1, Gpx4* and *Sod2* in the liver from the above groups. (I) mRNA levels of inflammatory factors *Il‐1β*, *Il‐6*, *Mcp‐1*, *Tnf‐α* and *Tgf‐β* in the liver from the above groups. (J) Hepatic levels of NAD^+^ and NADH in *Fut2^△IEC^* mice with and without hepatocyte overexpression of NAMPT. AAV‐B TBG vector: *N* = 5, AAV‐TBG *Nampt*: *N* = 7. n.s.: no significant difference.

As the hepatic NAD⁺ salvage pathway was disrupted in *Fut2^△IEC^* mice, an adeno‐associated virus that specifically expressed NAMPT in hepatocytes (AAV‐TBG *Nampt*) was constructed (Figure 6A). Hepatic steatosis and hepatocyte injury did not differ between AAV‐TBG *Nampt*‐treated *Fut2^△IEC^* mice and AAV‐TBG vector‐treated *Fut2^△IEC^* mice, as measured by H&E and Oil Red O staining, along with serum ALT and AST levels (Figure 6B–D). Serum LPS (Figure 6E) and ethanol (Figure 6F) were the same between *Fut2^△IEC^* mice treated with AAV‐TBG vector and AAV‐TBG *Nampt*. Liver lipid peroxidation similarly increased in AAV‐TBG *Nampt*‐treated *Fut2^△IEC^* mice and AAV‐TBG vector‐treated *Fut2^△IEC^* mice (Figure 6G). Although *Nrf2* expression was notably elevated in *Fut2^△IEC^* mice subjected to AAV‐TBG *Nampt* in comparison with those receiving AAV‐TBG vector, the expression levels of *Gpx1, Gpx4* and *Sod2* remained unchanged in the two groups (Figure 6H). The expression of hepatic inflammatory cytokines, including *Il‐1β*, *Il‐6*, *Mcp‐1*, *Tnf‐α* and *Tgf‐β*, showed no significant differences between the AAV‐TBG vector and AAV‐TBG *Nampt* treatment groups (Figure 6I). The hepatic NAD^+^ and NADH levels, and NAD^+^/NADH ratio did not differ markedly between groups (Figure 6J). These data imply that recovery of NAM‐mediated NAD^+^ synthesis could not compensate for the shortage of NAD^+^ and could not improve alcohol‐induced oxidative damage in the liver.

### 3.7 *Bifidobacterium* could not improve the metabolism of nicotinamide or the hepatic NAD^+^ synthesis

As *Bifidobacterium* was inversely associated with NAM and positively linked to PncA levels (Figure S5), we first investigated whether supplementing *Bifidobacterium* has the potential to enhance hepatic NAD**^+^** synthesis and alleviate alcohol‐induced liver damage. Previous studies have shown that 1,2‐fucosylated carbohydrates are the essential adhesion receptor of *Bifidobacterium*,^23^ so we used WT mice instead of *Fut2^△IEC^* mice.

Faecal differential metabolites were predominantly involved in phenylalanine metabolism and arginine biosynthesis pathways (Figure S6A), whereas those in the liver were associated with galactose metabolism and glycine, serine and threonine metabolism pathways (Figure S6C). Faecal levels of NA increased after supplementation with *Bifidobacterium aureus* (Figure S6B). NAM was detected only in the faeces of mice treated with PBS; thus, no comparative analysis was conducted. There was no difference in hepatic NAM between the PBS and *B. aureus* groups (Figure S6D). Neither group exhibited detectable hepatic levels of NA. Hepatic NAD⁺ levels tended to increase in mice administered *B. aureus* versus those given PBS; however, the difference was not significant (Figure S6E). Additionally, serum ALT levels exhibited a downward trend, but the change was not statistically significant (Figure S6F). Therefore, alternative strategies are needed to modulate bacterial metabolism of nicotinamide and restore hepatic NAD⁺ synthesis.

### 3.8 PncA restored hepatic NAD^+^ biosynthesis and alleviated alcoholic liver injury

**FIGURE 7:**
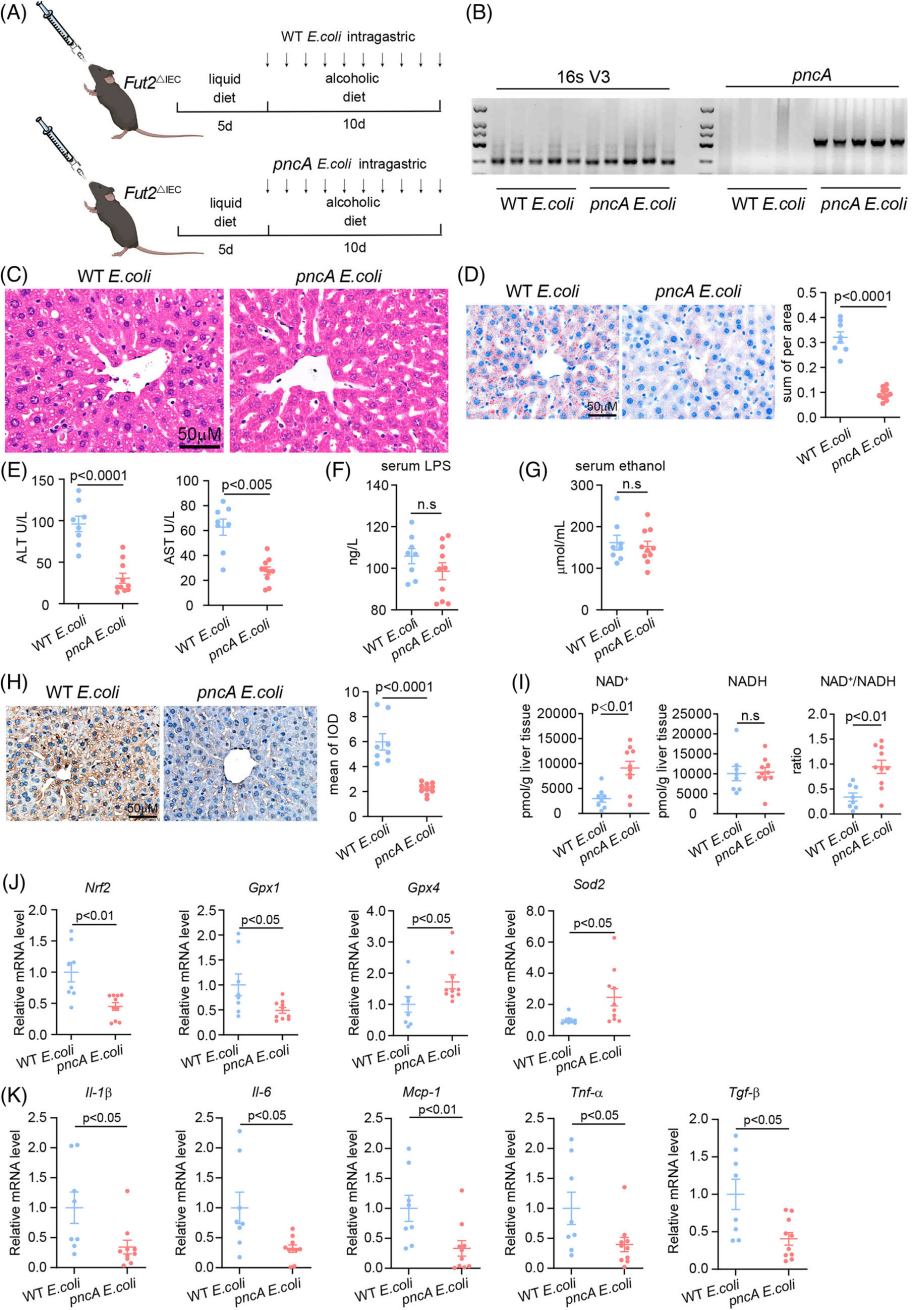
*pncA* overexpressed *Escherichia coli* ameliorated alcohol‐induced liver damage in Fut2^△IEC^ mice. (A) The strategy for intragastric administration of *E. coli* overexpressed *pncA* (*pncA E. coli*) in alcohol‐exposed Fut2^△IEC^ mice. (B) Faecal *pncA* abundances of alcohol‐exposed Fut2^△IEC^ mice treated with WT *E. coli* and *pncA* *E. coli* measured by PCR (*n* = 5). (C and D) Haematoxylin and eosin (H&E) staining (C) and Oil Red O staining (D) of hepatic tissues in Fut2^△IEC^ mice after intragastric administration of *pncA* *E. coli* (*n* = 10) or WT *E. coli* (*n* = 8). (E) Serum levels of ALT and AST in the above two groups. (F and G) Serum levels of LPS (F) and ethanol (G) of the above two groups. (H) Immunohistochemical staining of 4‐HNE in the liver tissues of the above two groups. (I) Hepatic levels of NAD^+^ and NADH in Fut2^△IEC^ mice treated with *pncA* *E. coli* (*n* = 10) and WT *E. coli* (*n* = 8). (J) Comparison of mRNA levels of oxidative stress‐related enzymes Nrf2, Gpx1, Gpx4 and Sod2 in the liver from the above groups. (K) mRNA levels of inflammatory factors *Il‐1β*, *Il‐6*, *Mcp‐1*, *Tnf‐α* and *Tgf‐β* in the liver of Fut2^△IEC^ mice from the above groups. n.s.: no significant difference.

*E. coli* overexpressing *pncA* (*pncA E. coli*) was constructed and administered to *Fut2^△IEC^* mice via gavage (Figure 7A). The *pncA E. coli* could colonise the intestines of *Fut2^△IEC^* mice (Figure 7B). Treatment with *pncA E. coli* significantly reduced hepatic steatosis and hepatocyte injury in alcohol‐exposed *Fut2^△IEC^* mice (Figure 7C–E). Serum LPS (Figure 7F) and ethanol levels (Figure 7G) were comparable between *Fut2^△IEC^* mice treated with WT *E. coli* and *pncA E. coli*. The level of 4‐HNE was decreased by *pncA E. coli* treatment (Figure 7H). Liver NAD⁺ level and the NAD⁺/NADH ratio were elevated in *Fut2^△IEC^* mice treated with *pncA E. coli* (Figure 7I). Hepatic *Nrf2* and *Gpx1* expression decreased in alcohol‐exposed *Fut2^△IEC^* mice treated with *pncA E. coli*, and *Gpx4* and *Sod2* increased in alcohol‐exposed *Fut2^△IEC^* mice treated with *pncA E. coli* (Figure 7J). Furthermore, mRNA levels of *Il‐1β*, *Il‐6*, *Mcp‐1*, *Tnf‐α* and *Tgf‐β* were significantly reduced in mice orally administered *pncA E. coli* compared to those given WT *E. coli* (Figure 7K). These data indicate that overexpression of PncA could promote hepatic NAD^+^ synthesis and alleviate alcoholic liver injury in *Fut2^△IEC^* mice.

## 4 DISCUSSION

NAD^+^ levels have been documented to decline under various pathological conditions, including ageing, obesity, inflammatory disorders, cardiovascular issues and neurodegenerative diseases.^24^ Excessive alcohol intake disrupts the NAD^+^/NADH ratio and causes oxidative stress in the liver.^25^ Restoring liver NAD^+^ levels has been reported to improve glucose homeostasis and mitochondrial dysfunction, and to effectively treat obesity and alcoholic and non‐alcoholic steatohepatitis.^26^ We found that the absence of intestinal epithelial α1,2‐fucosylation impaired bacterial deamination of nicotinamide, leading to reduced hepatic NAD⁺ synthesis via the Preiss–Handler pathway.

It has been reported that supplementation with NAD⁺ precursors exerts positive effects on blood lipid profiles and cholesterol, as well as provides short‐term benefits for type 2 diabetes.^27^, ^28^ NR has been recognised as an NAD^+^ precursor with a conserved metabolism across various organisms, and its presence in milk provides a dietary source for NAD⁺ biosynthesis. Chronic supplementation with NR has been demonstrated to elevate NAD⁺ levels in both liver and muscle tissues.^29^ Supplementation with NR could restore hepatic NAD^+^ and prevent alcohol‐related fatty liver.^15^, ^16^ The main route of oral NR to host NAD^+^ is via its conversion into NA by the gut microbiome.^19^ In addition to the de novo pathway, microbiota‐produced NA‐synthesised NAD^+^ contributes to 85% of the hepatic NAD^+^ pool.^30^ Hepatic uptake of NAD⁺ precursors mainly occurs as NA; oral administration of NAM and NR was significantly less effective at increasing hepatic NAD⁺ levels in *Naprt* knockout mice.^19^, ^31^ Oral administration of NA markedly alleviated alcohol‐induced liver injury in *Fut2* ^△IEC^ mice (Figure S7). Therefore, bacteria‐dependent NA production is essential for the NAD^+^ pool in the liver. These findings indicated that reinstating bacterial PncA expression, which is responsible for metabolising NAD^+^ precursors to synthesise NA in *Fut2* ^△IEC^ mice, confers resistance to alcohol‐induced oxidative stress damage by elevating NAD^+^ expression.

Studies have shown that hepatic *Nampt* deficiency exacerbates dyslipidemia, hepatic damage in high‐fat diet‐fed mice.^32^ Specific knockout of *Nampt* in hepatocytes disrupted NAD^+^ homeostasis in the livers of mice.^33^ We found that hepatocyte‐specific overexpression of NAMPT failed to restore hepatic NAD⁺ homeostasis or alleviate alcohol‐induced oxidative stress in *Fut2* ^△IEC^ mice. Restoring PncA expression in gut bacteria alleviates alcohol‐induced liver injury in *Fut2* ^△IEC^ mice by enhancing hepatic NAD⁺ synthesis. These findings further underscore the essential role of the Preiss–Handler pathway in hepatic NAD⁺ synthesis, and highlight the importance of gut bacteria in mammalian NAD⁺ metabolism. Our study suggests that in individuals with ALD and *FUT2* variants (non‐secretor status), oral supplements with NAM or NR combined with gut bacterial regulation may offer therapeutic benefits.

The *pncA* genes are commonly expressed in Firmicutes, Proteobacteria and Actinobacteria and are relatively low in Bacteroidetes.^34^ We observed a marked decrease in Firmicutes and Actinobacteria abundance of *Fut2* ^△IEC^ mice compared to controls (Figure 3C). This further confirmed the impairment of bacterial deamination of NAM in *Fut2* ^△IEC^ mice. The findings could reveal the damage caused by bacterial deamination of NAM in *Fut2* ^△IEC^ mice. In this study, we used engineered *E. coli* to restore intestinal NA levels in *Fut2* ^△IEC^ mice. Restoration of the NAD⁺ pool was demonstrated to alleviate alcohol‐related liver injury.

In summary, our study revealed that intestinal epithelial‐specific deletion of *Fut2* markedly reduced the abundance of bacterial *pncA*, disrupting nicotinamide metabolism and subsequently diminishing the hepatic NAD⁺ pool. This makes the liver susceptible to alcohol‐induced fat deposition and inflammation. For FUT2 non‐secretors, NAD^+^ precursor supplementation, such as NR and NAM, may not be as effective in preventing alcoholic liver injury as expected. One limitation of our study is the absence of genotyping data for the *FUT2* variant in patients with alcohol misuse and alcoholic hepatitis. To address this, we are currently expanding the sample size and assessing the ALD risk in *FUT2* non‐secretors.

## AUTHOR CONTRIBUTIONS

H.C., S.Y. and X.H. designed the study. L.C., Z.Y. and L.S. performed experiments. W.G., H.Y., SH.W. and SY.W. helped build the animal models and collect the patient data. L.C. drafted the manuscript. H.C., S.Y. and X.H. edited the manuscript. All the authors approved the final version of the manuscript.

## CONFLICT OF INTEREST STATEMENT

The authors declare no conflicts of interest.

## ETHICS STATEMENT

The animal experiments were approved by the Ethics Committee of the Union Hospital, Tongji Medical College, Huazhong University of Science and Technology (Approval No. 4242). Samples collected from patients were processed under approved protocols by the Research Ethics Committee of Union Hospital, Tongji Medical College, Huazhong University of Science and Technology (Approval No. [2022]0061‐01).

## Supporting information



Supporting Information

## Data Availability

The data are available from the corresponding author upon request.
