# Supplementary material for: Intestinal Fut2 deficiency exacerbated alcohol‐related liver disease by disrupting nicotinamide metabolism
Source: Clin Transl Med. 2025 Aug 15;15(8):e70447. doi: 10.1002/ctm2.70447 (PMC12356993; doi:10.1002/ctm2.70447)
Supplement: Supplementary file 1 — Supporting Information [file CTM2-15-e70447-s001.docx]

**Intestinal *Fut2* deficiency exacerbated alcohol-related liver disease by disrupting nicotinamide metabolism**

**Liuying Chen^#1,2^, Zhongwei Yin^#3^, Luorui Shang^#1^, Hang Yuan^2^, Wenkang Gao^2^, Shuhan Wang^1^, Shuyan Wang^2^, Xiaohua Hou*^2^, Shenglan Yang*^1^,** **Huikuan Chu*^2^**

^1^ Department of Clinical Nutrition, Union Hospital, Tongji Medical College, Huazhong University of Science and Technology, Wuhan, China.

^2^ Division of Gastroenterology, Union Hospital, Tongji Medical College,

Huazhong University of Science and Technology, Wuhan, China

^3^ Division of Cardiology and Hubei Key Laboratory of Genetics and Molecular Mechanisms of Cardiological Disorders, Tongji Hospital, Tongji Medical College, Huazhong University of Science and Technology, Wuhan, China

^#^ The authors contributed equally to this article.

* Correspondences: Huikuan Chu, [2012xh0827@hust.edu.cn](mailto:2012xh0827@hust.edu.cn); Shenglan Yang, [yangshenglan@126.com](mailto:yangshenglan@126.com); Xiaohua Hou, houxh@hust.edu.cn

**Supplementary methods**

**Animal experiments**

For mice subjected to *Bifidobacterium aureus* intervention during alcohol feeding, a daily oral gavage of 3×109 *Bifidobacterium aureus* was administered. The control groups were gavaged with phosphate-buffered saline (PBS).

**Targeted metabolome analysis**

The fecal and hepatic tissue samples of mice treated with *Bifidobacterium aureus* and PBS were separated using an Agilent 1290 Infinity LC ultra-high-performance liquid chromatography (UHPLC) system with HILIC and C18 columns. Mass spectrometry analysis was conducted using an AB 6500+ QTRAP mass spectrometer (AB SCIEX). The MRM raw data were processed using MultiQuant software for peak extraction to obtain the ratio of peak areas of each substance to the internal standard peak area. Content was calculated using a standard curve.

**PCR Amplification Conditions for *pncA***

PCR amplification was performed with an initial denaturation at 98°C for 5 minutes, followed by 40 cycles of denaturation at 95°C for 15 seconds, annealing at 65°C for 30 seconds, and extension at 72°C for 30 seconds per kilobase of the target amplicon.

**Supplementary tables and figures**

Supplemental table 1. Primers used in RT-PCR.

| Gene name | Forward sequence | Reverse sequence |
| --- | --- | --- |
| Mouse 18S | GTAACCCGTTGAACCCCATT | CCATCCAATCGGTAGTAGCG |
| Mouse *Il-1β* | TTGTTGATGTGCTGCTGTGA | TGTGAAATGCCACCTTTTGA |
| Mouse *Tgf-β* | GGCACCATCCATGACATGAACC | CCGCACACAGCAGTTCTTCTCT |
| Mouse *Il-6* | CTGCAAGAGACTTCCATCCAG | AGTGGTATAGACAGGTCTGTTGG |
| Mouse *Tnf-α* | CTGAACTTCGGGGTGATCGG | GGCTTGTCACTCGAATTTTGAGA |
| Mouse *Mcp-1* | TTAAAAACCTGGATCGGAACCAA | GCATTAGCTTCAGATTTACGGGT |
| Mouse *Nrf2* | TCTTGGAGTAAGTCGAGAAGTGT | GTTGAAACTGAGCGAAAAAGGC |
| Mouse *Gpx1* | AGTCCACCGTGTATGCCTTCT | GAGACGCGACATTCTCAATGA |
| Mouse *Gpx4* | GATGGAGCCCATTCCTGAACC | CCCTGTACTTATCCAGGCAGA |
| Mouse *Sod1* | AACCAGTTGTGTTGTCAGGAC | CCACCATGTTTCTTAGAGTGAGG |
| Mouse *Sod2* | CAGACCTGCCTTACGACTATGG | CTCGGTGGCGTTGAGATTGTT |
| Mouse *Nmnat1* | TGGGGCCAATGAGAGCAAG | CAACCCTCTGACAGCGATGTT |
| Mouse *Nmnat3* | CCTGTGGTTCCTTCAACCCC | AGATGATGCCCTCAATCACCT |
| Mouse *Naprt* | TGCTCACCGACCTCTATCAGG | CGAAGGAGCCTCCGAAAGG |
| Mouse *Nadsyn* | GTGGGGATGCCTATAATGCAC | TCCCGGTAGTTGCCTTCGT |
| Mouse *Nampt* | GCAGAAGCCGAGTTCAACATC | TTTTCACGGCATTCAAAGTAGGA |

Supplemental table 2. Primers used in PCR amplification of fecal pncA.

| Gene name | Forward sequence | Reverse sequence |
| --- | --- | --- |
| 16s V3 | TCGTCGGCAGCGTCAGATGTGTATAAGAGACAGCCAGACTCCTACGGGAGGCAG | GTCTCGTGGGCTCGGAGATGTGTATAAGAGACAGCGTATTACCGCGGCTGCTG |
| *pncA* | CGCTAACCGCCTGATTGACTG | ATTCACGCCACGACAACCATC |


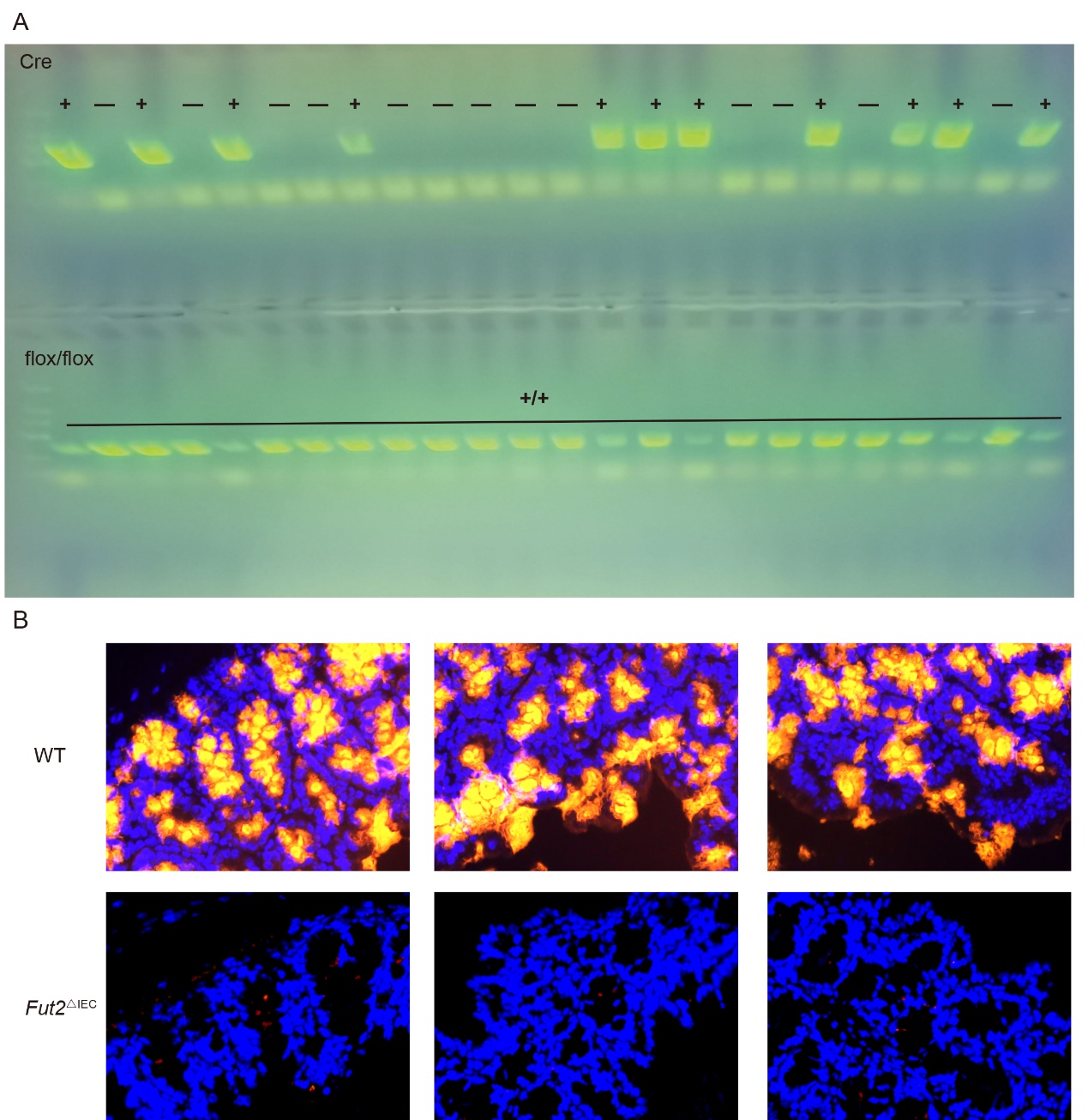


Supplemental figure 1. The efficiency and specificity of *Fut2* gene deletion in the mice colon. (A) Genotyping test by qPCR. (B) UEA-I staining of colonic tissues.


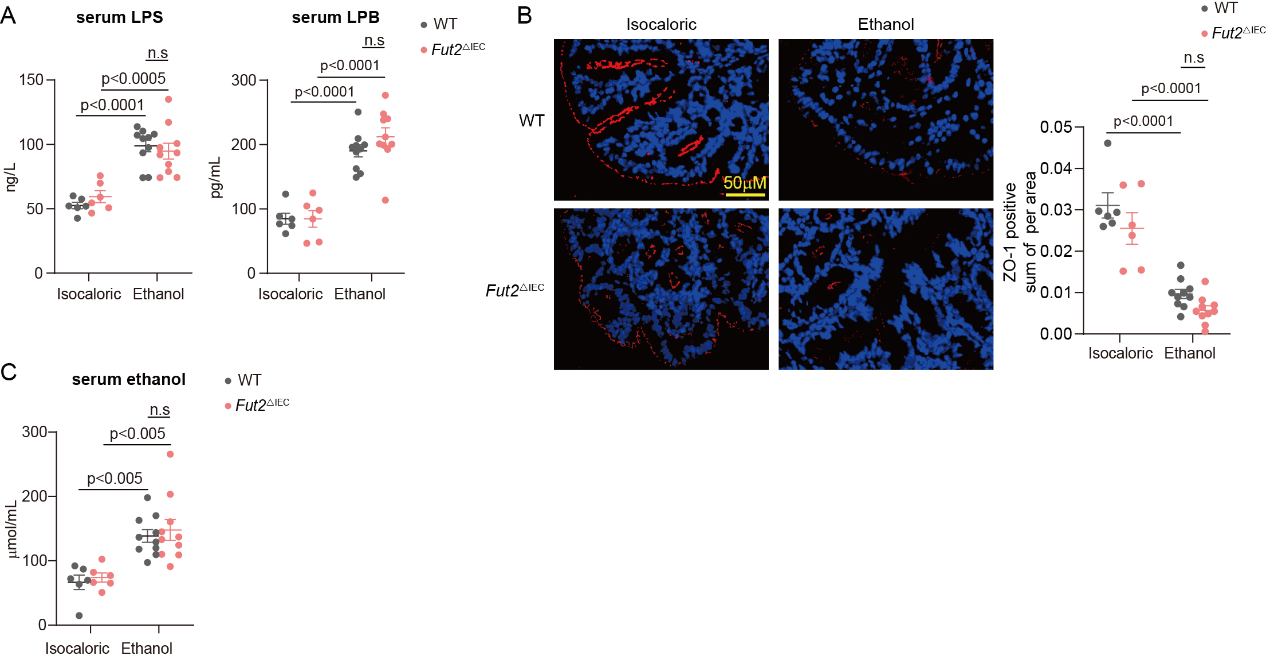


Supplemental figure 2. Changes in intestinal barrier function of wild-type and *Fut2^△IEC^* mice following alcohol exposure. (A) Serum LPS and LPB levels in control and alcohol-fed *Fut2^△IEC^* and WT mice. (B) Colonic ZO-1 immunofluorescence staining in wild-type and *Fut2^△IEC^* mice. (C) Serum ethanol levels in control and alcohol-fed *Fut2^△IEC^* and WT mice. n.s, no significant difference.


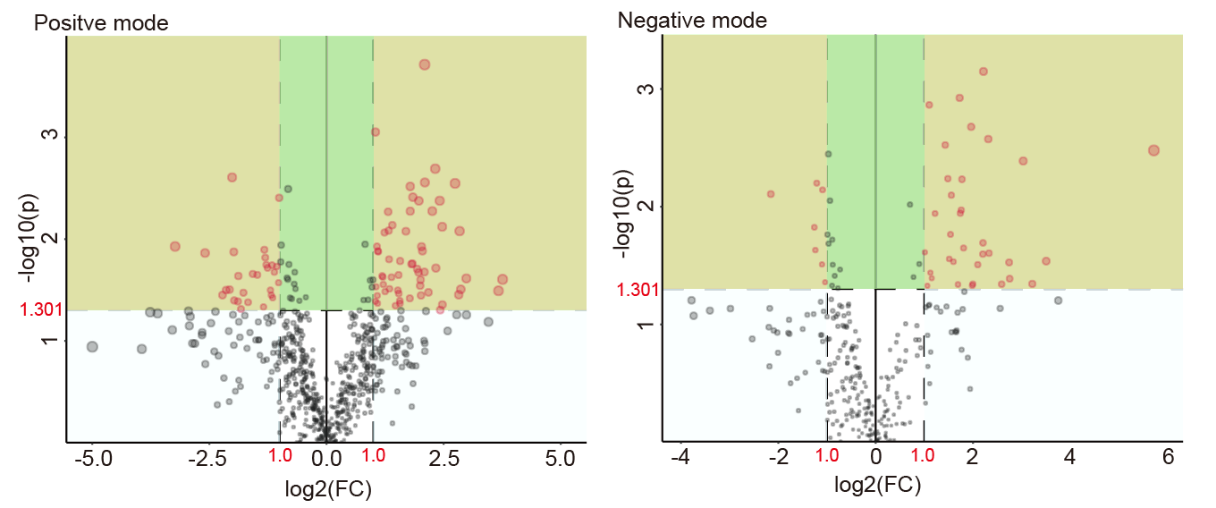


Supplemental figure 3. Volcano plots of different metabolites in positive and negative ion modes (|log_2_ (FC)|>1, p<0.05).


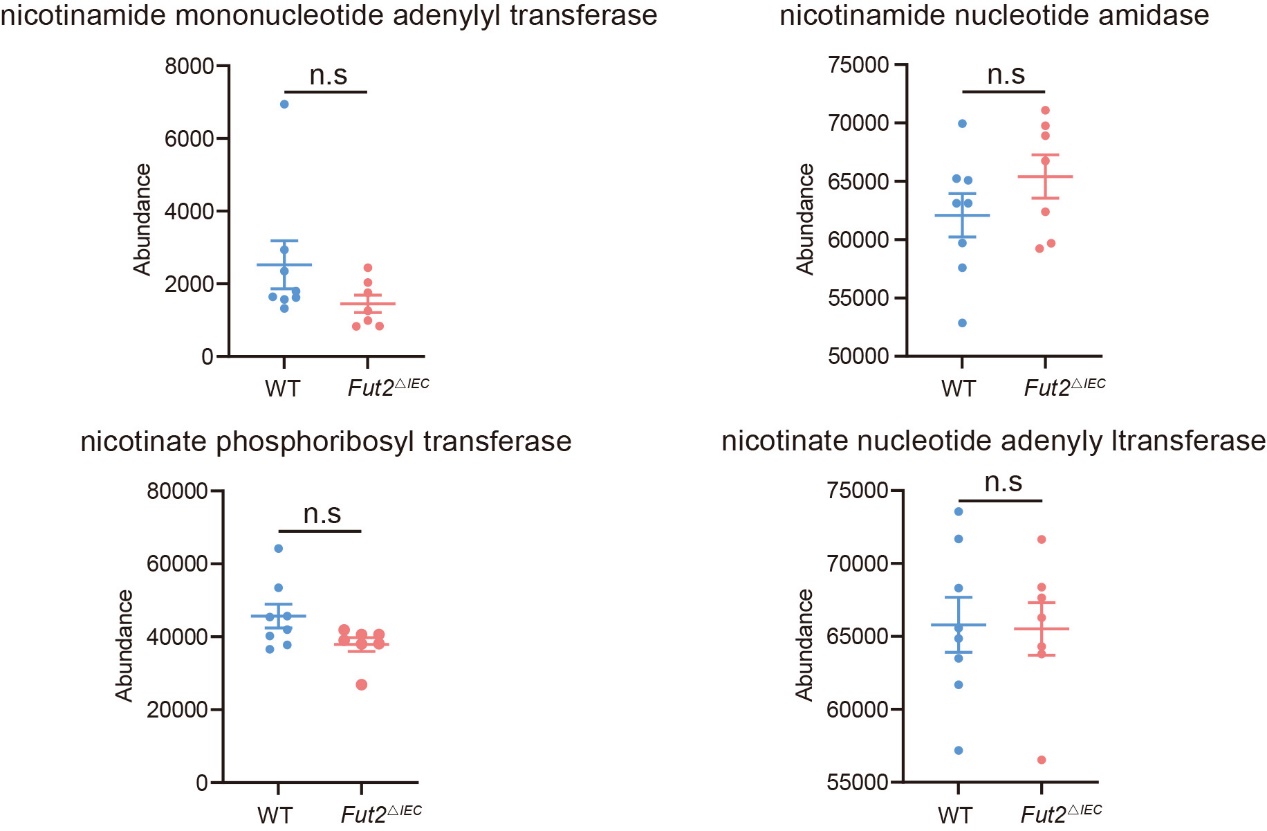


Supplemental figure 4. Comparison of the abundance of bacterial NAD^+^ synthetases between *Fut2^△IEC^* mice and WT mice. n.s, no significant difference.


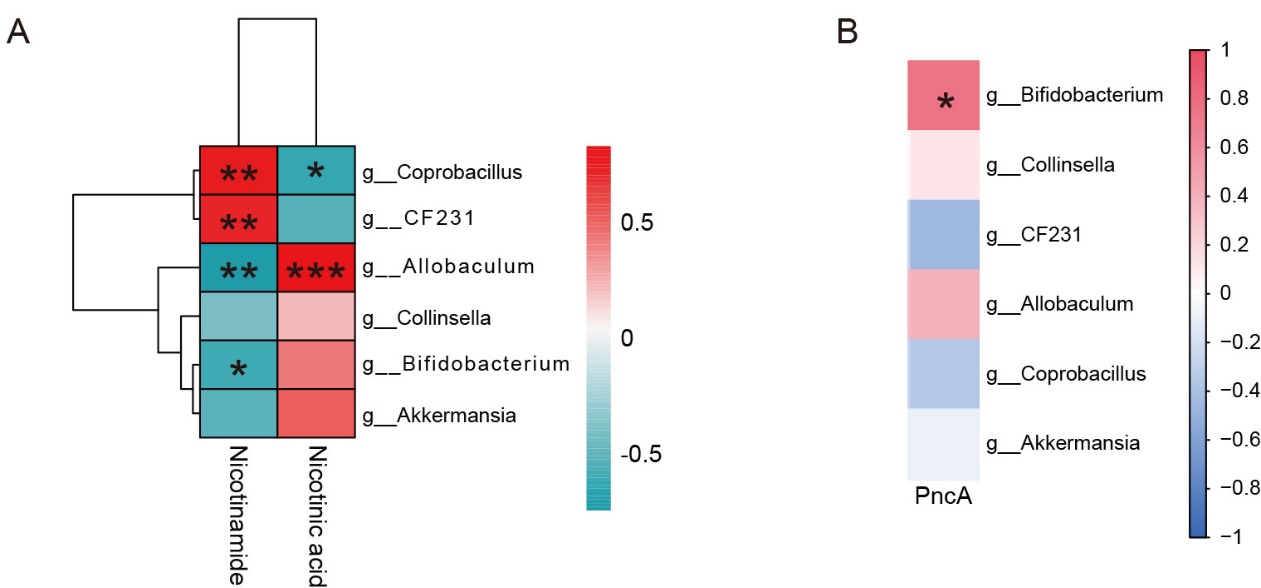


Supplemental figure 5. (A) Relationship among different bacterial genus and nicotinamide and nicotinic acid measured by spearman correlation. * indicated p<0.05, ** indicated p<0.01, *** indicated p<0.001. (B) Relationship between different bacterial genus and PncA levels using person correlation. * indicated p<0.05.


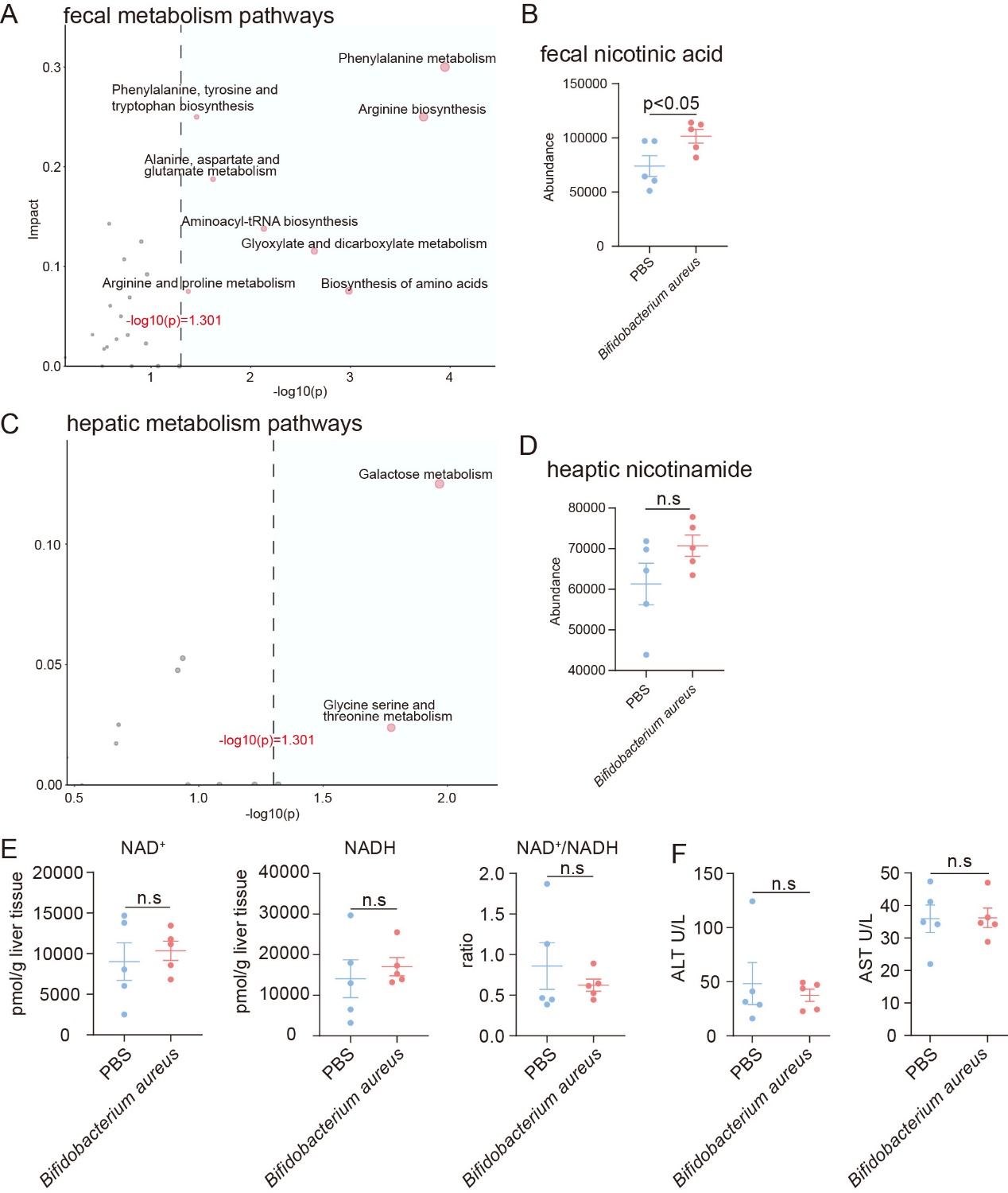


Supplemental figure 6. *Bifidobacterium* supplementation could not increase NAD^+^ expression in liver tissue of alcohol-fed mice. KEGG signaling pathways that enriched in by differential metabolites from feces (A) and liver tissues (C) of alcohol-exposed mice supplemented with *Bifidobacterium aureus* and PBS. (B) Comparison of nicotinic acid in feces of alcohol-exposed mice supplemented with *Bifidobacterium aureus* and PBS. (D) Comparison of nicotinamide in livers of alcohol-exposed mice supplemented with *Bifidobacterium aureus* and PBS. (E) Comparison of NAD^+^, NADH, and the ratio of NAD^+^/NAD in livers of alcohol-exposed mice supplemented with *Bifidobacterium aureus* and PBS. (F) serum ALT and AST levels of alcohol-exposed mice supplemented with *Bifidobacterium aureus* and PBS. B, *Bifidobacterium aureus*; n.s, no significant difference.


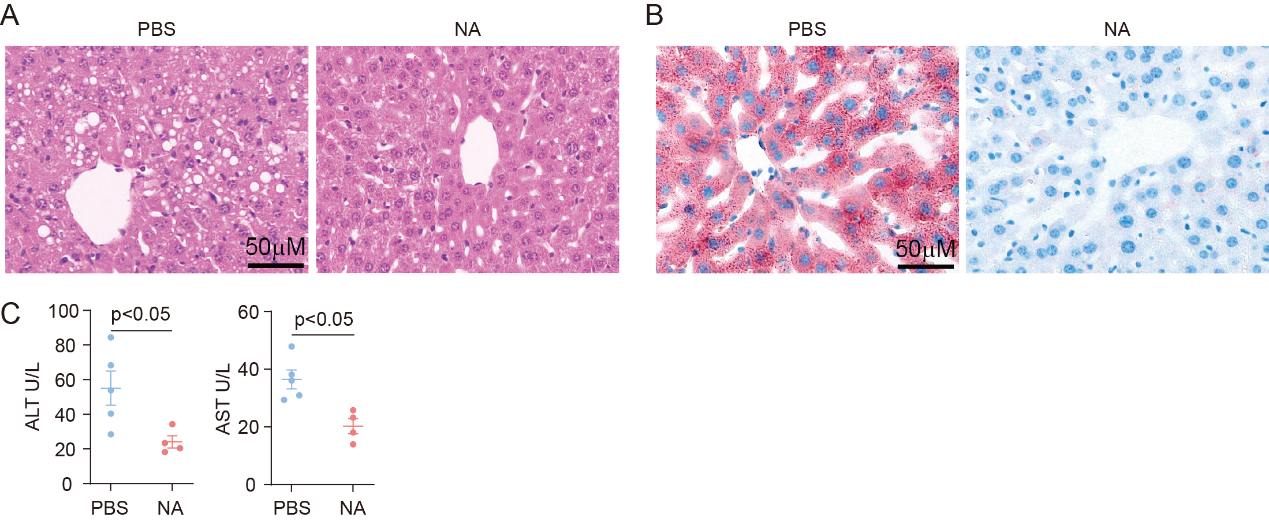


Supplemental figure 7. Nicotinic acid supplementation ameliorated alcoholic liver injury in *Fut2^△IEC^* mice. (A) H&E staining of liver tissues from *Fut2^△IEC^* mice treated with PBS or nicotinic acid (NA). (B) Oil Red O staining of liver tissues from above group. (C) Serum ALT and AST levels from above group.
